# DC-SIGN and Lung Pathogenesis in Patients with Tuberculosis

**DOI:** 10.1371/journal.pmed.0020410

**Published:** 2005-11-15

C-type lectins are carbohydrate-binding cell surface molecules with a wide range of biological functions, many of which are related to immunity. Despite its name, dendritic cell–specific intercellular adhesion molecule-3 grabbing nonintegrin (DC-SIGN) is not only expressed on dendritic cells but also on specialized macrophages in the placenta and lung. A number of pathogens are known to interact with DC-SIGN, and some (including HIV) seem to have evolved to derive advantages from these interactions.

Recent in vitro studies have shown that DC-SIGN can interact with *Mycobacterium tuberculosis* through a lipoglycan (a molecule composed of sugars and fatty acids) on the mycobacterial envelope called lipoarabinomannan (LAM). Trying to understand the role of DC-SIGN in tuberculosis (TB), Ludovic Tailleux and colleagues have focused on the interaction between *M. tuberculosis* and DC-SIGN–expressing cells in the lungs of human patients.

The researchers studied a total of 74 individuals, including 40 with TB, 11 with sarcoidosis, 14 with asthma, and nine control participants without active lung infection or inflammation. All patients underwent bronchoalveolar lavage (BAL), a procedure that yields cells and proteins from the lower respiratory tract. The researchers then examined BAL cell populations after staining for various cell-surface markers by flow cytometry, and found that, in individuals without TB, very few alveolar macrophages (an average of 3%) expressed DC-SIGN. In contrast, an average of 30% (and up to 70%) of macrophages from patients with TB expressed the lectin.

Tailleux and colleagues then incubated alveolar macrophages from a patient without TB ex vivo with *M. tuberculosis*, which resulted in infection of a subset of the cells. When the researchers examined DC-SIGN expression, they found that both infected and noninfected (bystander) cells in the population started to express DC-SIGN. The effect on bystander cells suggests that soluble factors from the microbe and/or the infected cells can induce DC-SIGN expression. Further functional ex vivo studies with cells from human patients indicated that DC-SIGN expression renders alveolar macrophages more susceptible to infection.

The authors propose a scenario where complement receptors mediate most of the initial infection of alveolar macrophages in a naïve host, and where—once the infection is established—DC-SIGN–expressing alveolar macrophages become preferential target cells for *M. tuberculosis*. Future work will be focused on identifying the soluble factors involved, and on determining whether DC-SIGN induction is an essential part of TB pathogenesis.

